# Quality of life and wellbeing among HIV outpatients in East Africa: a multicentre observational study

**DOI:** 10.1186/s12879-014-0613-1

**Published:** 2014-11-18

**Authors:** Richard Harding, Victoria Simms, Suzanne Penfold, Julia Downing, Eve Namisango, Richard A Powell, Faith Mwangi-Powell, Scott Moreland, Nancy Gikaara, Mackuline Atieno, Irene J Higginson

**Affiliations:** Department of Palliative Care, Policy & Rehabilitation, Cicely Saunders Institute, King’s College London, Bessemer Road, London, SE5 9RJ UK; London School of Hygiene and Tropical Medicine, Keppel St, London, WC1E 7HT UK; African Palliative Care Association, Plot 850 Dr Gibbons Road, Kampala, Kampala, Uganda; Global Health and Knowledge Management Consultant, London, UK; Formerly APCA and Currently in USAID|ASSIST, University Research Company, Nairobi, Kenya; Futures Group, MEASURE Evaluation, University of North Carolina, Chapel Hill, USA

**Keywords:** HIV, Quality of life, Self-report, Sub-Saharan Africa, Mental health

## Abstract

**Background:**

Global health investment has reduced HIV mortality and transmission. However, little is known of patient-reported outcomes alongside ART rollout. This study aimed to measure wellbeing using patient-reported outcome measures (PROMS) among outpatients at PEPFAR-funded facilities.

**Methods:**

In a multicentre 2 country cross-sectional study, adults attending 12 facilities in Kenya and Uganda gave self-reported data on quality of life (physical and mental wellbeing dimensions), functional and a measure of multidimensional problems (physical, psychological, social and spiritual).

**Results:**

Among the 1,337 participants, multidimensional problems were more common in psychological, spiritual and social domains than in physical. In multivariable analysis using GEE to adjust for facility effect, the mental health subscale of quality of life was lower for people with limited functional status (B = -5.27, 95% CI -5.99, 1. -4.56 p < 0.001) and higher for wealthier people (B = 0.91, 95% CI 0.48, 1.33, p < 0.001). The physical health subscale of quality of life was lower for those with limited functional status (B = -8.58, 95% CI -9.46 to -7.70, p < 0.001) and those who had a caregiver present (B = -1.97, 95% CI -3.72 to -0.23, p = 0.027), higher for wealthier people (B = 1.14, 95% CI 0.65, 1.64, p < 0.001), and positively associated with CD4 count (B = 1.61, 95% CI 1.08-2.14, p < 0.001). Multidimensional problems were more burdensome for people with limited functional status (B = -2.06, 95% CI -2.46 to -1.66, p < 0.001), and less burdensome with more education (B = 0.63, 95% CI 0.25-1.00, p = 0.001) or ART use (B = 0.94, 95% CI 0.34-1.53, p = 0.002).

**Conclusions:**

Multidimensional problems are highly prevalent, and worse with declining function. Importantly, ART use does not appear to be protective for self-reported physical and mental dimensions of quality of life. Assessment and management of self-reported wellbeing must form part of HIV care and treatment services to ensure maximum benefit from ART investment.

## Background

High prevalence and associated burden of physical and psychological problems have been reported among HIV outpatients with access to antiretroviral therapy (ART) in high income countries [1]-[4]. Within the generalised epidemic of sub-Saharan Africa, evidence of patient-reported problems since the rollout of ART has mainly related to later stages of HIV disease [5],[6]. The World Health Organisation (WHO) definition of health is ‘complete physical, mental and social well-being’, [7] and WHO guidance also identifies the need for the assessment and management of physical, psychological, social and spiritual problems from the point of HIV diagnosis and alongside treatment [8].

Although global evidence suggests that patients bear a high burden of problems from the point of HIV diagnosis [9], clinical skills and research since the advent of ART have focused mainly on outcomes of viral suppression and delaying mortality, and arguably have lost patient-centredness [10]-[12]. Patient reported outcome measures (PROMS) have been identified as essential tools to promote quality and equity in healthcare [13]. An evaluation of the effect of PEPFAR (President’s Emergency Plan for AIDS Relief) funding in its target countries has established that there has been a decrease in HIV-related deaths [14] and a reduction in the number of HIV-positive births [15]. However, there has been a lack of evaluative evidence of the management of patients’ health from their own perspective; therefore it is unclear whether optimal benefit is being achieved from treatment investment.

This paper aims to measure patient wellbeing using PROMS among HIV outpatients at PEPFAR-funded facilities, and to determine associations with patient problems.

## Methods

### Study design

As part of a mixed-methods Public Health Evaluation (PHE) of the President’s Emergency Plan for AIDS Relief (PEPFAR) Care and Support programme, a multi-centre, longitudinal quantitative study was conducted of care received and patient-health outcomes over three months in Kenya and Uganda (full protocol available [16]). This paper presents the cross-sectional analysis of results at baseline.

### Study design and setting

During the longitudinal observational cohort of the PHE, consecutive HIV-infected outpatients were recruited from 12 PEFAR-funded care facilities in Kenya and Uganda. Kenya and Uganda are PEPFAR focus countries with generalised epidemics.

### Ethics

Ethical approval to undertake the study was received from the Uganda National Council for Science and Technology (UNCST, Ref SS 1964), the Kenyan Medical Research Institute (Ref KEMRI/RES/7/3/1) and the College Research Ethics Committee at King’s College London (Ref CREC/06/07-140). Subsequent tool changes following initial piloting were also approved.

### Procedure for recruitment, data collection and analysis

Adult outpatients who were aware of their HIV diagnosis and able to give informed consent (either written or thumb print depending on literacy level) were invited into the study. Consecutive patients were approached in outpatient waiting areas in the order of their attendance, and trained researchers conducted consenting and data collection. All questionnaires were forward/back translated into local languages, and all questions read aloud. Respondents gave a verbal response to each item, and the study researcher recorded the response. This reduced any potential bias, as limited literacy could lead to a mix of self-completion and researcher-completion. Patients gave a self-report on demographic data as follows; age (coded 18-29, 30-39, 40-63), gender (male/female), country of recruitment (Kenya/Uganda), number of dependents (coded as 0-1, 2-3, 4+), time to travel to the facility (coded as an hour or less/more than an hour), and ART use (yes/no). Socioeconomic status was measured following the methodology used in the Demographic and Health Surveys [17], using variables including house construction, possession of items, fuel supply etc. The entire sample of 1,337 people was included. ART use and CD4 count were the only variables not self-reported (these were extracted from file).

Finally three self-report scales were completed, each having been previously validated in sub-Saharan African populations. These were: the Medical Outcome Scale - HIV (MOS-HIV), which is a 35-item quality of life scale in Uganda and widely used in Africa [18],[19] consists of two subscales of physical and mental health (each subscale analysed as quintiles) and is sensitive to treatment effects [20]; the APCA African Palliative Outcome Scale (POS) [21] has seven patient-reported multidimensional items that reflects the WHO definition of palliative care, (i.e. it measures physical, psychological, social and spiritual problems among those with incurable life-limiting illness, analysed as quintiles, and referred to as the POS); the ECOG Performance Status, which is a single item rating of functional status ranging from 0 = fully active to 5 = dead. (coded 0/1/2+) [22]. Participants were paid $5 for travel expenses.

### Data management and entry

Data were collected in a private space away from other patients, and questionnaires carried only a unique study participant identifier. Questionnaires were stored in a locked cabinet. Data were entered into a pre-designed EpiData v3.1 database (Odense Denmark: EpiData Association; 2000-2008). Errors were identified using consistency checks and double-entry validation, and followed-up by manual checking of questionnaires. Stata v10 (Texas: Stata Corp LP; 2007) was used to undertake descriptive analysis and tests of association. MOS-HIV item scores were converted into continuous summary scores for mental and physical health (0-100) [23]. The scores for the seven patient-completed items of the APCA African POS (pain, symptoms, worry, peace/spiritual wellbeing, sharing feelings, feeling life worthwhile, help and advice) were summed to create a total patient POS score. Additionally, all individual POS item scores were coded into ternary variables (0/1 = low problem, 2/3 = moderate problem, 4/5 = severe problem) presented by facility. The wealth quintile variable was created following the methods of the Demographic Health Surveys [17],[24], using factor analysis with principal components analysis to create a continuous variable. This variable was then divided into quintiles. Age was grouped into categories: 18-29, 30-39, 40-49, 50-59 and 60-70. CD4 was categorised 0-100, 101-200, 201-350 and 351 to maximum. Demographic characteristics and outcomes were described by facility (facilities were labelled A-M to retain anonymity). The association between continuous outcomes (mental health, physical health and total POS score) and facility was tested using analysis of variance.

The main outcomes were quality of life using two subscales of physical and mental health score from the MOS-HIV, and the POS [23].

T-tests and analysis of variance were used to identify demographic and clinical characteristics associated with the three outcomes in bivariate analysis. The individual POS item scores were included as co-variates for quality of life mental and physical health subscales but not for total POS scores, because they were components of it and so association was inevitable. Multilevel fixed-effects linear regression was used to analyse whether scores were associated with personal characteristics, adjusting for facility clustering. All variables associated with an outcome in bivariate analysis were taken forward into the multivariate model. CD4 count was not used in a model if the presence of a CD4 test result on file was associated with the outcome. Population-averaged models were fitted using generalised estimating equations (GEE) to adjust for the effect of facility. The three outcomes were mental health subscale score, physical health subscale score, and total patient POS score.

## Results

### Sample description

Table 1 shows the demographic characteristics of the sample by facility. One participant did not give demographic data, leaving a total sample in the analysis of 1336 participants, an average of 111.4 per facility (range 102-125). In total 68.3% were female (57.7%-78.5% by facility), and the mean age was 34.8 (32.1-38.8 by facility).

**Table 1:** **Demographic characteristics of sample by facility N = 1336**

		Facility											
		A	B	C	D	E	F	G	H	J	K	L	M
***N***		*109*	*111*	*120*	*120*	*125*	*111*	*107*	*105*	*112*	*107*	*102*	*107*
**Gender (% female)**		68.8	74.8	65.8	72.5	70.4	57.7	78.5	64.8	60.7	61.7	71.6	72.0
**Mean age (range)**		33.0 (18-69)	38.6 (21-63)	33.9 (18-59)	33.5 (18-58)	34.8 (19-58)	33.7 (18-60)	37.8 (18-70)	38.8 (21-66)	32.1 (18-53)	33.7 (22-67)	34.9 (18-55)	33.0 (19-61)
**Education**	None	5.6	2.7	1.7	5.0	2.4	0.9	25.2	14.3	4.5	3.7	6.9	8.4
	Began primary	69.4	54.1	55.8	50.0	40.8	54.1	53.3	57.1	35.7	58.0	37.3	53.3
	Began secondary	22.2	38.7	30.8	37.5	46.4	37.8	20.6	25.7	48.2	26.2	39.2	27.1
	Diploma	1.9	3.6	10.8	7.5	7.2	6.3	0.9	1.0	6.3	10.3	13.7	8.4
	Degree	0.9	0.9	0.8	0	3.2	0.9	0	1.9	5.4	1.9	2.9	2.8
**Wealth quintile**	Poorest	39.5	2.7	5.0	1.7	15.2	37.3	80.4	43.8	2.7	3.7	5.9	19.6
	Middle poor	32.1	27.0	25.8	3.3	14.4	24.6	6.5	47.6	4.5	10.3	8.8	26.2
	Middle	19.3	33.3	15.8	15.0	33.6	19.1	10.3	4.8	19.6	23.4	15.7	27.1
	Middle wealthy	8.3	19.8	21.7	28.3	21.6	12.7	1.9	2.9	42.9	30.8	30.4	17.8
	Wealthiest	0.9	17.1	31.7	51.7	15.2	6.4	0.9	1.0	30.4	31.8	39.2	9.4
**Has a CD4 test result on file (%)**		88.1	91.0	85.8	99.2	90.4	89.2	100.0	41.0	50.4	46.7	90.2	33.6
**CD4 count (%)**	**N**	96	101	103	116	113	99	107	43	57	50	92	36
	**0-100**	16.7	2.0	29.1	27.6	13.3	15.2	9.4	9.3	12.3	16.0	20.7	8.3
	**101-200**	15.6	19.8	19.4	19.8	17.7	24.2	30.8	7.0	10.5	20.0	16.3	13.9
	**201-350**	28.1	37.6	20.4	21.6	29.2	21.2	36.5	30.2	22.8	36.0	22.8	36.1
	**351+**	39.6	40.6	31.1	31.0	39.8	39.4	23.4	53.5	54.4	28.0	40.2	41.7
**Newly diagnosed (%)**		36.7	2.7	63.3	54.2	19.2	35.1	0	17.1	70.8	25.2	34.3	29.0
**Taking ART (%)**		66.7	67.6	8.3	40.0	56.5	31.5	91.6	36.2	8.9	14.0	38.2	60.7
**Has a carer (%)**		8.3	9.0	15.8	12.5	14.4	10.8	98.1	3.8	13.4	3.7	19.7	9.4
**ECOG**	0	56.0	74.8	80.0	75.0	58.4	71.2	20.6	79.1	82.1	51.4	43.1	23.4
	1	33.9	22.5	19.2	20.8	34.4	20.7	37.4	19.1	9.8	43.9	45.1	63.6
	2	9.2	2.7	0.8	4.2	6.4	7.2	38.3	0	4.5	2.8	8.8	8.4
	3 or 4	0.9	0	0	0	0.8	0.9	3.7	1.9	3.6	1.9	2.9	4.7
**Mean number of care components received during study (SD)**		15.5 (6.0)	11.5 (6.2)	8.4 (4.9)	15.8 (6.7)	10.1 (5.8)	9.9 (5.1)	23.9 (4.2)	22.0 (6.3)	13.0 (6.4)	13.8 (3.9)	20.1 (4.1)	16.0 (4.3)

### Descriptive outcomes

For the entire sample of n = 1336, mean quality of life mental health subscale score was 46.2 (95% CIs 45.7-46.8) (Table 2). By facility, the range was from 40.3 to 49.7 and the difference between facilities was statistically significant (F = 10.46, p < 0.001). Quality of life physical health subscale mean score was 44.9 for the entire sample (95% CIs 44.2-45.5), ranging from 37.9 to 49.5 by facility, which was statistically significant (F = 12.61, p < 0.001). The mean total POS score was 20.8 (95% CIs 20.5-21.1) and ranged from 19.1 to 22.6 by facility, which was also statistically significant (F = 6.94, p < 0.001).

**Table 2:** **Patient outcome scores by facility, MOS-HIV and POS N = 1336**

		A	B	C	D	E	F	G	H	J	K	L	M	All
**Mean mental health score (SD)**		47.0 (10.1)	49.7 (8.2)	47.5 (10.2)	48.4 (9.5)	49.5 (9.7)	46.4 (8.6)	42.6 (7.3)	40.3 (8.9)	43.9 (10.6)	47.0 (9.4)	47.7 (9.3)	43.9 (9.6)	**46.2 (9.7)**
**Mean physical health score (SD)**		43.1 (12.8)	49.5 (9.2)	47.4 (11.6)	46.7 (11.8)	47.1 (9.6)	42.6 (11.2)	37.9 (9.7)	38.5 (12.3)	49.0 (11.4)	45.8 (10.9)	46.7 (12.5)	42.8 (10.7)	**44.9 (11.7)**
**POS: Mean total patient score (SD)**		22.6 (5.5)	22.1 (4.9)	20.9 (5.5)	22.4 (4.9)	20.6 (5.1)	22.0 (5.8)	21.3 (4.9)	19.2 (4.4)	19.7 (4.3)	20.0 (5.5)	19.6 (4.5)	19.1 (5.3)	**20.8 (5.2)**
**Pain (%)**	Moderate	47.7	43.2	35.8	31.7	53.6	47.8	71.0	68.6	33.9	38.3	51.0	59.8	**48.2**
	Severe	8.3	6.3	4.2	8.3	14.4	6.3	15.9	18.1	2.7	14.0	2.9	15.0	**9.7**
**Symptoms**	Moderate	36.7	26.1	40.8	20.8	33.6	35.1	58.9	50.4	35.7	48.6	36.3	36.5	**38.1**
	Severe	4.6	1.8	0.8	3.3	4.0	1.8	9.4	4.8	0.9	9.4	2.0	8.4	**4.2**
**Worry**	Moderate	22.9	20.7	30.0	29.2	30.4	32.4	57.0	61.0	42.9	16.8	42.2	35.5	**34.9**
	Severe	17.4	9.0	10.0	11.7	9.6	9.9	15.0	16.2	13.4	24.3	6.9	14.0	**13.0**
**Difficulty sharing feelings**	Moderate	31.2	27.0	24.2	19.2	21.6	21.6	31.8	32.4	32.1	27.1	65.7	33.6	**30.2**
	Severe	47.7	49.6	66.7	60.8	55.2	45.1	6.5	15.2	61.6	40.2	33.3	31.8	**43.5**
**Difficulty finding life worthwhile**	Moderate	15.6	14.4	20.0	12.5	16.8	13.5	23.4	59.1	31.3	20.6	36.3	43.0	**25.1**
	Severe	3.7	23.4	10.0	9.2	12.8	21.6	0.9	17.1	11.6	6.5	7.8	22.4	**12.3**
**Lack of peace**	Moderate	28.4	21.6	30.8	30.0	28.0	22.5	42.1	62.9	48.2	21.5	49.0	45.8	**35.6**
	Severe	11.0	13.5	15.8	13.3	26.4	13.5	33.6	23.8	23.2	33.6	11.8	34.6	**21.1**
**Need for help/advice**	Moderate	22.9	22.5	14.2	17.5	27.2	47.8	48.6	31.4	14.3	21.5	32.4	34.6	**27.7**
	Severe	36.7	59.5	69.2	52.5	51.2	36.9	10.3	41.9	77.7	71.0	65.7	44.9	**51.6**

Review of the POS scores shows that items for which the greatest proportion of respondents fell into the “severe problem” category were ‘need for help/advice’ (51.6% severe) and ‘difficulty sharing feelings’ (43.5% severe) and feeling at peace (21.1%). In seven facilities, more than half of participants had severe problems with obtaining help/advice. For both these items there was only one facility where fewer than 10% had severe problems - facility G. Psychological, spiritual and social problems were more common than physical problems.

### Bivariate analysis

At 5% significance, quality of life mental health subscale was associated with wealth quintile, education, having a caregiver present, functional status and having a CD4 count on file, while physical health was associated with age group, wealth quintile, education, ART use, CD4 count result, being newly diagnosed, having a carer present and functional status (Table 3). Poorer and less educated people, with limited functional status, those with a carer present and those lacking a CD4 count on file were more likely to have lower (worse) mental health subscale score. Older, poorer and less educated people taking ART, with a long-standing HIV diagnosis, those having a carer present and those with limited function were more likely to have lower (worse) physical health. Both the MOS-HIV outcomes were associated with CD4 count result, but since having a CD4 count was associated with mental health, the CD4 test result could not be considered as a covariate of mental health because of sampling bias. Total patient POS score was associated with wealth quintile, education, ART use, functional status and having a CD4 count on file. POS multidimensional problems were more common among people who were poorer, less educated, with lower function, who were not taking ART or did not have a CD4 count on file.

**Table 3:** **Bivariate analysis for each outcome MOS-HIV physical health, MOS-HIV mental health, and POS N = 1336**

		Mental health score		Physical health score		Total patient POS score	
		Mean (95% CIs)	Test result	Mean (95% CIs)	Test result	Mean (95% CIs)	Test result
**Gender**	Male	46.6 (45.7-47.5)	t = 0.94, p = 0.347	44.1 (42.9-45.2)	t = -1.64, p = 0.101	21.0 (20.5-21.6)	F = 1.15, p = 0.284
	Female	46.1 (45.4-46.7)		45.2 (44.5-46.0)		20.7 (20.4-21.0)	
**Age group**	18-29	46.3 (45.2-47.3)	F = 1.11, p = 0.348	45.9 (44.7-47.1)	F = 2.64, p = 0.032	20.7 (20.2-21.2)	F = 1.71, p = 0.146
	30-39	45.9 (45.1-46.7)		45.1 (44.2-46.1)		20.5 (20.1-20.9)	
	40-49	46.4 (45.3-47.5)		43.3 (41.8-44.7)		21.3 (20.7-21.9)	
	50-59	48.2 (46.4-50.1)		43.7 (41.1-46.2)		21.6 (20.6-22.5)	
	60-70	44.5 (40.3-48.7)		42.0 (39.1-44.9)		22.1 (19.0-25.2)	
**Wealth quintile**	Poorest	43.1 (42.1-44.2)	F = 14.41, p < 0.001	40.2 (38.8041.5)	F = 25.26, p < 0.001	20.8 (20.1-21.4)	F = 2.48, p = 0.042
	Middle poor	45.3 (44.1-46.6)		42.9 (41.5-44.4)		20.4 (19.8-21.0)	
	Middle	46.4 (45.2-47.5)		45.2 (43.8-46.5)		20.6 (19.9-21.2)	
	Middle wealthy	47.7 (46.6-48.8)		47.6 (46.3-48.8)		20.6 (20.0-21.2)	
	Wealthiest	48.8 (47.6-50.0)		48.7 (47.3-50.1)		21.7 (21.1-22.3)	
**Education**	None	43.6 (41.6-45.5)	F = 4.65, p = 0.003	41.0 (38.5-43.5)	F = 5.65, p < 0.001	19.6 (18.5-20.7)	F = 5.31, p = 0.001
	Began primary	46.1 (45.4-46.9)		44.4 (43.5-45.2)		20.7 (20.3-21.0)	
	Began secondary	46.3 (45.4-47.2)		45.9 (44.9-46.9)		20.9 (20.4-21.4)	
	Diploma+	48.7 (47.1-50.3)		46.7 (44.2-49.1)		22.4 (21.4-23.3)	
**ART**	Yes	46.4 (45.7-47.2)	t = -0.62, p = 0.536	44.0 (44.7-46.4)	t = 2.34, p = 0.020	21.3 (20.9-21.8)	F = 10.59, p = 0.001
	No	46.1 (45.4-46.8)		45.5 (43.1-44.9)		20.4 (20.0-20.8)	
**Newly diagnosed**	Yes	46.4 (45.5-47.3)	t = -0.46, p = 0.648	46.3 (45.1-47.4)	t = -3.09, p = 0.002	20.8 (20.4-21.3)	F = 0.01, p = 0.936
	No	46.2 (45.5-46.8)		44.2 (43.4-44.9)		20.8 (20.5-21.1)	
**Has a carer**	Yes	43.5 (42.4-44.7)	t = 4.82, p < 0.001	39.9 (38.3-41.4)	t = 7.48, p < 0.001	21.2 (20.5-21.8)	F = 1.50, p = 0.220
	No	46.8 (46.3-47.4)		46.0 (45.3-46.6)		20.7 (20.4-21.0)	
**Functional status**	0	49.0 (48.4-49.7)	F = 72.73, p < 0.001	49.7 (49.0-50.4)	F = 197.63, p < 0.001	21.8 (21.5-22.2)	F = 32.01, p < 0.001
	1	43.1 (42.2-43.9)		39.8 (38.8-40.8)		19.6 (19.1-20.1)	
	2	39.4 (38.0-40.9)		31.7 (30.0-33.5)		18.1 (17.1-19.1)	
	3/4	35.7 (33.2-38.2)		24.3 (20.9-27.7)		17.7 (15.7-19.7)	
**Has a CD4 test result on file**	Yes	46.9 (46.3-47.4)	t = -4.16, p < 0.001	45.1 (44.4-45.8)	t = -1.41, p = 0.159	21.0 (20.7-21.3)	F = 7.10, p = 0.008
	No	44.3 (43.2-45.4)		44.1 (42.7-45.4)		20.1 (19.6-20.7)	
**CD4 count**	0-100	49.0 (48.2-49.9)	F = 8.33, p < 0.001	44.5 (43.3-45.6)	F = 25.41, p < 0.001	20.7 (19.9-21.5)	F = 0.38, p = 0.769
	101-200	50.4 (49.8-51.1)		47.6 (46.8-48.4)		21.2 (20.4-21.9)	
	201-350	51.4 (50.8-51.9)		49.8 (49.2-50.4)		21.2 (20.6-21.8)	
	351-max	51.3 (50.9-51.8)		50.5 (50.0-51.0)		21.0 (20.5-21.5)	
**Pain**	Low	50.1 (49.4-50.8)	F = 99.40, p < 0.001	51.3 (50.5-52.1)	F = 217.20, p < 0.001		
	Moderate	44.2 (43.5-44.9)		41.4 (40.5-42.2)			
	Severe	39.8 (38.0-41.6)		34.4 (32.6-36.3)			
**Symptoms**	Low	48.8 (48.2-49.5)	F = 78.98, p < 0.001	49.2 (48.4-49.9)	F = 165.51, p < 0.001		
	Moderate	43.2 (42.4-43.9)		39.7 (38.8-40.7)			
	Severe	38.6 (36.0-41.2)		32.3 (29.5-35.1)			
**Worry**	Low	49.9 (49.2-50.5)	F = 136.9, p < 0.0016	47.9 (47.1-48.6)	F = 54.81, p < 0.001		
	Moderate	43.5 (42.7-44.3)		42.4 (41.3-43.5)			
	Severe	39.1 (37.6-40.7)		39.6 (37.9-41.3)			
**Sharing feelings**	Low	45.4 (44.4-46.4)	F = 6.10, p = 0.002	41.9 (40.7-43.2)	F = 41.51, p < 0.001		
	Moderate	45.5 (44.6-46.4)		42.8 (41.6-43.9)			
	Severe	47.3 (46.5-48.1)		48.1 (47.2-48.9)			
**Finds life worthwhile**	Low	48.1 (47.5-48.8)	F = 49.89, p < 0.001	46.0 (45.2-46.8)	F = 12.06, p < 0.001		
	Moderate	42.3 (41.3-43.2)		42.3 (41.1-43.5)			
	Severe	44.6 (43.0-46.2)		44.3 (42.4-46.1)			
**Peace**	Low	51.0 (50.3-51.7)	F = 169.79, p < 0.001	48.1 (47.2-49.0)	F = 49.65, p < 0.001		
	Moderate	43.9 (43.1-44.7)		43.6 (42.5-44.6)			
	Severe	40.3 (39.2-41.4)		40.3 (38.9-41.7)			
**Help and advice**	Low	48.6 (47.5-49.7)	F = 10.27, p < 0.001	45.3 (43.9-46.7)	F = 5.30, p = 0.005		
	Moderate	45.8 (44.8-46.7)		43.2 (42.0-44.4)			
	Severe	45.6 (44.8-46.3)		45.6 (44.7-46.4)			

All seven patient-completed POS items were associated with both the MOS-HIV outcomes. Mental and physical health quality of life subscale scores were lower (worse) for participants with moderate or severe pain, symptoms and worry, and for those who reported low scores for finding peace or for obtaining help and advice. Participants with moderate problems finding life worthwhile had slightly lower physical and mental health scores than those with severe difficulty. Participants with severe difficulty sharing feelings had better MOS-HIV scores than those with low or moderate problems.

### Multivariable analysis

Using GEE (Table 4), mental health quality of life subscale was lower (worse) for people with limited functional status (B = -5.27 95% CI -5.99, -4.56) and slightly higher (better) for wealthier people (B = 0.91, 95% CI 0.48 to 1.33, p < 0.001). Physical health quality of life subscale was lower (worse) for those with limited functional status (B = -8.58, 95% CI -9.46 to -7.670, p < 0.001) and for those who had a caregiver present (B = -1.97, 95% CI -3.72 to -0.23, p = 0.027), higher (better) for wealthier people (B = 1.14, 95% CI 0.65 to 1.64, p < 0.001), and positively associated with CD4 count (B = 1.61, 95% CI 1.08-2.14, p < 0.001). Multidimensional POS problems were more burdensome for people with limited functional status (B = -2.06, 95% CI -2.46 to -1.66, p < 0.001), less burdensome for those with more education (B = 0.63, 95% CI 0.25-1.00, p = 0.001) or those using ART (B = 0.94, 95% CI 0.34-1.53, p = 0.002).

**Table 4:** **Generalised estimating equations for each model MOS-HIV physical health, MOS-HIV mental health, and POS N = 1336**

	Mental health score		Physical health score		POS score	
	Coefficient (95% CIs)	p	Coefficient (95% CIs)	p	Coefficient (95% CIs)	p
**Age**			-0.05 (-1.08,0.01)	0.079		
**Wealth quintile**	0.91 (0.48,1.33)	<0.001	1.14 (0.65,1.64)	<0.001	0.16 (-0.08,0.39)	0.192
**Functional status**	-5.27 (-5.99,-4.56)	<0.001	-8.58 (-9.46,-7.70)	<0.001	-2.06 (-2.46,-1.66)	<0.001
**Newly diagnosed**			-0.08 (-1.47,1.30)	0.907		
**Has a carer**	-1.13 (-2.63,0.37)	0.141	-1.97 (-3.72,-0.23)	0.027		
**CD4 count**			1.61 (1.08,2.14)	<0.001		
**Education**	0.07 (-0.60,0.74)	0.838	0.11 (-0.69,0.92)	0.783	0.63 (0.25,1.00)	0.001
**Using ART**			1.00 (-0.29,2.23)	0.128	0.94 (0.34,1.53)	0.002

## Discussion and conclusion

Our data reveal severe problems for patients’ access to help and advice and to their ability to share feelings. Generally, social and spiritual problems were self-reported as worse than physical problems. This suggests that the concept of health as defined by the WHO needs greater clinical attention. In the context of a generalised epidemic and limited resources, implying a high patient load per health professional, time may be limited for help and advice. Routine assessment of multi-dimensional problems and referral to non-medical/nursing staff may be a feasible approach to achieving wellbeing. Holistic assessment and care are required, especially as we found that those with worse physical and mental health reported poor wellbeing in terms of being at peace (a measure of spiritual wellbeing [25]), and gaining help and advice. It is also important to note that within the POS, the greatest proportion of respondents who identified severe problems were for the items on accessing help and advice, sharing feelings, and being at peace. Inequalities persist in terms of those with less education having a worse POS score (multidimensional problems) and worse mental health for the less wealthy, demonstrating that within the global health debate we must recognise inequalities within low income countries. The quantitative POS data are supported by qualitative data from these facilities, which identified the multidimensional problems of outpatients in line with the WHO definition of palliative care, i.e. problems in the physical, psychological, social and spiritual domains [26]. The data also demonstrate a higher burden of physical and mental ill-health, and multidimensional problems, with poorer functional status. Socioeconomic status and education are closely associated with each other, therefore only one or the other is associated with outcomes in multivariate models. We note from previous analysis of people newly diagnosed with HIV in East Africa that help/advice is closely associated with education (contributing significantly to the total POS score) [27], which may explain our finding of an association between education rather than poverty and total POS score.

An analysis of ART-na’ve HIV outpatients in Uganda found very similar MOS-HIV mean physical subscale score (46.18 vs 44.9 in our sample) and mental subscale score (46.19 vs 46.2) [28]. The MOS-HIV subscale mean scores in our sample are worse compared to recent data from Belgium (mean physical health subscale in Belgium 55.6 vs 44.9 in our sample, mean mental health 52.0 in Belgium vs 46.2 in our sample) [29] although a greater proportion of the sample in Belgium were male (78.9% vs 31.7% and on ART (92.0% vs 43.4%).

There are several limitations to our data. Firstly, the use of self-report data may be less robust in regions with limited literacy and less experience of questionnaire use. We reduced this potential bias by only using questionnaires validated on local populations, and by researchers administering all questionnaires. Second, clinical data (i.e. ART use and CD4 count) were reliant on availability on file, and therefore we were unable to analyse variables where this was not routinely collected and recorded. We have reported separate analyses of factors associated with the presence of a CD4 count on file [30]. Third, the data are cross sectional and therefore we can determine associations not causality.

It is notable that our findings confirm previous European evidence that ART use is not associated with physical or mental health [3]. We do not suggest that ART is not effective in improving outcomes, but that people on treatment continue to have multidimensional problems that require support across domains of need to achieve optimal health. We recently completed two trails of an intervention [31] in response to the findings in the present paper that these problems persist and are burdensome alongside treatment.

From these data we conclude that all patients require holistic assessment and care irrespective of ART use or functional status, but that those with worse function or with a lower socioeconomic status require additional support beyond the medical management of their HIV disease.
